# THE FAITH CARE FAMILY PROJECT: A QUALITATIVE ANALYSIS OF AN INTERVENTION FOR AFRICAN AMERICAN AD/ADRD CAREGIVERS

**DOI:** 10.1093/geroni/igae098.2280

**Published:** 2024-12-31

**Authors:** Noelle Fields, Ling Xu, Ishan Williams, Fayron Epps, Swasati Handique

**Affiliations:** The University of Texas at Arlington, Arlington, Texas, United States; The University of Texas at Arlington, Arlington, Texas, United States; University of Virginia, Charlottesville, Virginia, United States; The University of Texas Health San Antonio, San Antonio, Texas, United States; University of Texas at Arlington, Arlington, Texas, United States

## Abstract

Alzheimer’s disease and related dementias (AD/ADRD) disproportionately impact older African American populations. Although there are interventions for AD/ADRD caregiving, there remain research gaps related to culturally congruent psychoeducational interventions for African American AD/ADRD family caregivers. The Faith Care Family (FCF) Project was developed to train volunteers from a predominantly African American church to work with African American AD/ADRD family caregivers through a 6-week, telephone-based intervention. Qualitative interviews were conducted with the family caregivers (N = 9) following the intervention. Using a semi-structured interview guide, questions focused on knowledge of AD/ADRD, caregiver stress and coping, feelings about the behavioral symptoms of AD/ADRD, differences in caregiving before and after the FCF Project, and recommendations for future program iterations. Braun and Clarke’s six-step thematic analysis was followed to examine the interview data. The majority of caregivers identified as women, and the median age was 62. Over half had some college or below, and most reported little to no financial strain. The median length of caregiving was 5 years, and caregivers typically spent 10 hours per day providing care. Slightly over half of caregivers lived with the care recipient. Thematic analysis revealed four themes: establishing a connection, normalizing the caregiving experience, reinforcing knowledge, and resource sharing. Recommendations included adding additional AD/ADRD content to the manual. Overall findings suggest that the FCF Project is a promising intervention for African American AD/ADRD family caregivers. Lessons learned and recommendations for future research on ‘faith-placed’ ADRD interventions are offered.

